# Retraction Note: Neurotropic influenza A virus infection causes prion protein misfolding into infectious prions in neuroblastoma cells

**DOI:** 10.1038/s41598-025-00273-2

**Published:** 2025-05-06

**Authors:** Hideyuki Hara, Junji Chida, Keiji Uchiyama, Agriani Dini Pasiana, Etsuhisa Takahashi, Hiroshi Kido, Suehiro Sakaguchi

**Affiliations:** 1 Division of Molecular Neurobiology, Institute for Enzyme Research (KOSOKEN), Tokushima University, Kuramoto 3-18-15, Tokushima, 770-8503 Japan; 2 Division of Enzyme Chemistry, Institute for Enzyme Research (KOSOKEN), Tokushima University, Kuramoto 3-18-15, Tokushima, 770-8503 Japan

Retraction of: *Scientific Reports* 10.1038/s41598-021-89586-6, published online 12 May 2021

The Authors have retracted this Article.

In follow-up work to this Article, the Authors discovered that experiments using new stocks of the neurotropic influenza A virus strain A/WSN/33 (H1N1) failed to reproduce the findings reported in the Article regarding the conversion of PrPC into PrPSc in N2aC24 cells. The Authors, therefore, do not have confidence in the conclusions regarding the ability of the virus to cause misfolding of PrPC.

All Authors agree with this retraction.

